# Editorial comments to “Increased interleukin‐6 levels are associated with atrioventricular conduction delay in severe COVID‐19 patients”

**DOI:** 10.1002/joa3.13135

**Published:** 2024-08-21

**Authors:** Bonpei Takase, Nobuyuki Masaki

**Affiliations:** ^1^ Department of Intensive Care Medicine National Defense Medical College Tokorozawa Japan; ^2^ Division of Cardiology Iruma Heart Hospital Iruma Japan

**Keywords:** cardiovascular disease, cytokines, infectious disease

Many cardiovascular diseases including atherosclerotic ischemic heart diseases, chronic heart failure, and cerebrovascular disorders are associated with chronic and acute inflammatory activation.^1^, ^2^ In mechanisms of developing arrhythmias, inflammation has recently been reported as one of the important pathogenic factors. In this important and a novel finding has also been recognized as the mechanism of rhythm disturbance complicated with COVID‐19 infection.^3^ Especially, Interleukin‐6 has been focused because of possible influence to gap junction of connexin 40 or 43 which is important for the maintenance of normal heart rhythms.^4^, ^5^ In current issue of this journal, Accioli et al.^1^ reported the important role of Interleukin‐6 of COVID‐19 infection. The study is *for the first time* conducting prospective study on the role of Interleukin‐6 for developing atrioventricular conduction disturbance, even if the numbers of study population was small of 33 patients. Since development of heart rhythm disturbance in patients of COVID‐19 is sign for untoward outcome of COVID‐19, clarifying the mechanism of atrioventricular conduction disturbance and founding Interleukin‐6 possibly become treatment target is very important for treatment of long COVID‐19 and severe COVID‐19 patients. In this aspect, Accioli et al.'s findings should be confirmed in larger cohort.

Besides, COVID‐19 infection, Interleukin‐6 levels could be related with pathogenesis of HFpEF patients^2^ or other cardiovascular disorders so that Accioli et al. study could be incentive for further advance in the research of the role of Interleukin‐6 in the field of arrhythmia disease other than atrioventricular conduction disturbance.

## CONFLICT OF INTEREST STATEMENT

Authors declare no conflict of interests for this article.
